# Supplementary material for: Identifying differences between those with suicidal ideation-with-action, compared to ideation alone, using a community representative sample
Source: PLoS One. 2025 May 29;20(5):e0317163. doi: 10.1371/journal.pone.0317163 (PMC12121802; doi:10.1371/journal.pone.0317163)
Supplement: S1 Appendix — (DOCX) [file pone.0317163.s001.docx]

**Appendix 2: Sensitivity Analyses**

Sensitivity analyses were conducted to determine if important differences exist between the 2002 and 2012 surveys, since the study relies on pooling together respondents from these two surveys. Analyses were also conducted to examine the impact of excluding observations with missing data.

***Numbers of respondents self-reporting past-year suicidal ideation***

The 2012 survey included respondents who self-reported suicidal ideation during the worst episode of feeling depressed in the universe from which the question of past-year suicidal ideation was asked. The 2002 survey did not. However, the proportion of respondents that had past-year suicidal ideation from 2002 is 24% and this is comparable to the proportion who had past-year suicidal ideation from 2012 (27%) despite the 2002 survey not including the individuals with suicidal ideation during worst/bad episode into the universe for the past-year suicidal ideation question.

***Numbers of respondents reporting seeking professional help***

Among those with ideation, in the 2002 survey, the proportion of respondents who reported seeking professional help - which includes visiting ED, hospitalization, walk-in clinic, consulting a family doctor or psychologist in person or by telephone – was 9% (n=20) and in the 2012 survey, that proportion was 29% (n=47). This three-fold increase in proportion who sought help may indicate cultural trends that facilitate help-seeking in the 2012 sub-sample, such as de-stigmatization of mental problems, and is a factor to be mindful of when considering the pooled population of this study.

***Sociodemographic variables***

Ensuring the pooled population from the two surveys are not dissimilar in terms of sociodemographic variables is important since we know, for example, that sex is a factor in suicidal behaviour, with higher rates of ideation and attempts among females [1, 2]. In terms of sociodemographic variables, both cycles had similar proportions of respondents in terms of age, sex, marital, and income. In terms of mood and anxiety disorders, and in substance use disorders, 2002 and 2012 survey respondents had similar proportions.

None of the differences above were of concern in terms of affecting the final results of the study.

***Missing data***

Categorizing missing sociodemographic variables (age, sex, marital status, income, education and employment) as separate categories for analysis resulted in no meaningful differences. Where missing data were few, the cases were deleted from the analytic dataset, with no major impact to the result.

References

1. Borges G, Nock MK, Haro Abad JM, Hwang I, Sampson NA, Alonso J, et al. Twelve-month prevalence of and risk factors for suicide attempts in the World Health Organization World Mental Health Surveys. J Clin Psychiatry. 2010;71(12):1617-28. doi: 10.4088/JCP.08m04967blu.
2. Nock MK, Borges G, Bromet EJ, Alonso J, Angermeyer M, Beautrais A, et al. Suicidal ideation as a marker of psychological distress: Evidence from a large-scale community survey. Psychol Med. 2008;38(7):1045-56. doi: 10.1017/S0033291708002515.
